# Effect of Multi-sensory Stimulation on Neuromuscular Development of Premature Infants: A Randomized Clinical Trial

**Published:** 2018

**Authors:** Hossein ZERAATI, Fatemeh NASIMI, Akram REZAEIAN, Javad SHAHINFAR, Maryam GHORBAN ZADE

**Affiliations:** 1 Anesthesiology Department, School of Nursing and Midwifery, North Khorasan University of Medical Sciences, Bojnurd, Iran.; 2 Faculty of Nursing, Department of Nursing, Jahrom University of Medical Sciences, Jahrom,Iran; 3 Department of Pediatric Nursing, School of Nursing and Midwifery, Mashhad University of Medical Sciences, Mashhad, Iran; 4 Pediatric Department, School of Nursing and Midwifery, North Khorasan University of Medical Sciences, Bojnurd, Iran

**Keywords:** Multisensory-stimulation, Neurodevelopment, Premature infants, New Ballard scale

## Abstract

**Objectives:**

Preterm birth is considered as a risk factor for developmental disabilities, which can lead to long-term effects on the nervous system of children. The aim of this study was to determine the effect of multi-sensory stimulation on neurodevelopment of premature infants.

**Materials and Methods:**

In this two-group double-blind clinical trial in Jahrom Hospital, Jahrom, Iran from Jun to Aug 2016, 80 preterm infants were randomly divided. The intervention group received multisensory stimulation for 12 min per session, 5 sessions per wk along with routine NICU care and the control group received ward's routine care. Neuromuscular maturity for each infant was assessed by New Ballard Score. Data were analyzed using independent *t*-test.

**Results:**

Based on ANOVA with repeated measures, New Ballard score significantly changed in the intervention group before and after intervention (*P*= 0.001). This change was also significant in the control group (*P*=0. 04). However, the changes in New Ballard score were significantly different before and after intervention between the two groups (*P*=0.001).

**Conclusion:**

Multi-sensory stimulation can have beneficial effects on the development of neuromuscular in premature infants.

## Introduction

According to WHO, the infants born earlier than 37 wk from the first day of last menstruation are considered premature infants and its prevalence is estimated as 5%-7% globally (1). Premature birth is the most common cause of infants' death considered as one of the risk factors for developmental disabilities, which can lead to long-term complications in the nervous system of infants (2).

Following preterm birth, natural developmental processes are impaired, especially when the infant is born so early, which requires intensive care (3). They are at risk for developmental problems. The causes of developmental problems are first, their premature birth may be due to pre-existing problems, second, birth in such premature pregnancy can cause damage to vital organs like heart and lungs, and third, this damage can occur during the neonatal period following treatment measures (4).

In addition, exhaustion caused by compliance or stress can also lead to injury or damage to development. Moreover, very stimulatory environment of the hospital and lack of social interaction experiences with mother and great interaction with others can add to the above risks. Many reasons are conceivable that by itself or in interaction with other causes can lead to cause the problems related to the growth and development of premature infants. Often it is unclear how much premature infants are affected by these cases (5). Although the brain of the infants born at 25 to 40 wk, is still immature but is rapidly evolving (6). Impairment of brain growth and development in the early stages can affect some basic structures formed during this course of brain development, but there still needs to further evolve (7). All neuro-motor capacities should more evolve to be prepared for functions that are more complex after infant's discharge from the hospital (6, 7).

Developmental care is a broad classification of interventions designed concerning improving developmental outcomes in premature infants admitted to the neonatal intensive care unit (8). Multi-sensory stimulation is relatively a new intervention closely related to principles of evolutionary care (9). Since 1960, different researchers have proposed different types of multi-sensory stimulation for premature infants admitted in the hospital with aim to simulate the intrauterine environment at the first weeks of life in order to maintain and facilitate the development of premature infants (10).

Different stimulation programs included auditory touch-motor or situational stimulation or visual stimulation (11). Sensory stimulation, either single or multi-sensory stimulation, had positive outcomes and results in the process of evolutionary domains (12). Now, there is contradictory evidence of the effect of multi-sensory stimulation from neuromuscular aspect in premature infants and short-term effects of multi-sensory stimulation (auditory, tactile, vestibular and visual stimulation) on neuromuscular development in premature infants have rarely been reported (9-12).

The aim of this study was to determine the effect of multi-sensory stimulation on neuromuscular development of premature infants.

## Materials and Methods

This two-group double-blind randomized clinical trial study was conducted from Jun to Aug 2016. The studied population were preterm infants admitted to Jahrom Hospital, Jahrom central Iran who entered to the study based on information contained in the records.

This study was conducted with a confirmation ethics of Jahrom University of Medical Sciences (IRCT code: IRCT2016073114454N2).

Inclusion criteria of infants included: The preterm infants born between 32 and 36 wk of gestation, the infant has no history of cardiopulmonary resuscitation, the infant has no history of surgery, the infant's 5 min Apgar is not less than 6, the infant has no history of intraventricular hemorrhage grade 2 and above, the infant has no major congenital malformations. The exclusion criteria were medically unstable preterm infants.

Eighty preterm infants were obtained through a pilot study and formula of means' comparison. A minimum sample of 36 patients in each group was required based on a margin of error á = 0.05 and ß = 10%, expected power of 90%, and Z value of 1.28. However, we decided to enroll 40 patients in each group. The sample loss did not occur in this study.

Then, 80 preterms were randomly enrolled in either intervention and control groups (40 infants in each group) using random number table. They had an equal probability of being assigned to each of the two groups.

At first, the form of selecting subjects including the inclusion and exclusion criteria was completed by the researcher through interviews with parents of infants and the eligible infants were selected. Then, required explanation about the purpose of the study was given to the parents by the researcher as face-to-face. 

If they tend to participate in the study, written informed consent was obtained and the form of infants' individual characteristics was completed

In the intervention group multisensory stimulation was performed. The control group received the routine care in the NICU. The stimulation was started after 48 h of birth.

In the intervention group after stabilization of infant's condition, intervention including a multisensory stimulation program for duration 12 min daily, 5 d per wk until discharge from the hospital. The following multisensory stimuli were provided Auditory Stimulation —Soft lullaby between (30–40 dB) for 3 min using a miniature speaker; Tactile Stimulation—Gentle stroking massage for 3 min in a sequence of chest, upper limbs and lower limbs in supine position; Visual Stimulation—Black and white visual stimulation card hung at a distance of 8–10 in. from the neonate for 3 min; Vestibular Stimulation—Gentle horizontal and vertical rocking for 3 min.

The neuromuscular development was assessed before and after intervention. New Ballard scale was used as the outcome measure to assess the neuromotor development of the preterm (13). Ballard Scale is a commonly used neurological assessment. It assigns a score to various criteria (Posture, Square window, Arm recoil, Popliteal angle, Scarf sign, Heel to ear), the sum of all of then extrapolated to neuromuscular development. Four criteria are scored from 0 through 4, and one criterion is scored from 0 through 5 in the original Ballard Score. The scores were then ranged from 0 to 25.

The New Ballard scale in this study was assessed by inter-assessor reliability method. To follow this method, two trained colleagues were asked to record pain scores simultaneously in 10 cases in separated questionnaires. The correlation coefficient of 91% was obtained in this test. 

In order to lack of bias in the results of the study, who measure to assess the neuromotor development of the preterm by using the New Ballard Scale was blind to the groups.

Data were analyzed using SPSS software version 16 (Chicago, IL, USA). To evaluate the normal distribution of quantitative data, Kolmogorov-Smirnov and Shapiro–Wilk were used. In order to compare variables between the two groups, independent *t*-test was used in the case of normally distributed; to compare inter-groups dependent variables at different stages, variance analysis with repeated measures was used. *P*<0.05 was considered significant.

## Results

There was no significant difference between intervention and control groups in terms of gestational age, birth weight, height, length and head circumference and 1^st^ and 5^th^ min Apgar score; therefore, two groups are homogeneous in terms of this variable (*P*>0.05) (Table 1).

Table 2 shows New Ballard score increased 15.2±2.2 to 23.7±1.9 in intervention group and 14.8±2.5 to 18.1±2.4 in control group. *t*-test showed a significant difference between the two groups in terms of New Ballard scores after intervention (*P*=0.001).

The results of variance analysis with repeated measurements showed that New Ballard score significantly changed in the intervention group before and after intervention (*P*=0.001). This change was also significant in the control group (*P*=0.04). However, the changes in New Ballard score were significantly different at before and after intervention between the two groups (*P*=0.001) (Table 2).

Total score of neuromuscular development was 20.7±1.6 in the intervention group and 15.9±1.5 in the control group (Table 3). Mann-Whitney test showed significant relationship between two groups in terms of Posture, Arm Recoil, Popliteal angle, Heel to ear and Total of neuromuscular development with new Ballard tools.

**Table 1:** Baseline characteristics of participants with mean and standard deviation

Demographics	Intervention group		Control group		*t*-test
	Mean	SD	Mean	SD	
Gestational Age (wk)	32.1	2.0	32.8	1.8	*P*=0.58
Weight (g)	1400.1	250.4	1441.4	261.8	*P*=0.23
Length (cm)	38.2	2.5	39.0	2.7	*P*=0.32
Head circumference	28.0	1.5	28.6	1.3	*P*=0.66
Apgar score 1 min	6.4	2.7	6.1	2.5	*P*=0.51
Apgar score 5 min	8.8	1.7	8.6	2.2	*P*=0.59
Length of NICU stay (d)	21.3	3.9	22.1	4.2	*P*=0.37

**Table 2:** Comparison of the Mean Score of New Ballard score in Two Studied Groups

Neuromuscular Maturity	Intervention group		Control group		*t*-test
	Mean	SD	Mean	SD	
Before Intervention	15.2	2.2	14.8	2.5	*P*= 0.43
After intervention	23.7	1.9	18.1	2.4	*P*=0.001
The results of variance analysis with repeated measurements		Between-groups intervention		*P*=0.001	
		Between-groups Control		*P*=0.04	
		Comparison of two groups		*P*=0.001	

**Table 3:** Comparison of New Ballard Score components between control group and Intervention group

Neuromuscular Maturity	Intervention group		Control group		Mann Whitney U test
	Mean	SD	Mean	SD	
Posture	3.03	0.7	2.03	0.5	*P*<0.005
Square Window	3.2	0.4	3.03	0.8	*P*=0.334
Arm Recoil	3.2	0.7	2.4	0.6	*P*<0.005
Popliteal angle	3.9	0.7	2.9	0.9	*P*<0.005
Scarf sign	3.1	0.6	3.0	0.7	*P*=0.641
Heel to ear	3.3	0.5	2.5	0.8	*P*<0.005
Total	20.7	1.6	15.9	1.5	*P*<0.005

## Discussion

Due to the high cost of care for premature infants and their neurological and physiological problems, care after the birth of these infants has been considered by the researchers from last few decades to improve the living environment of these infants and effective and essential changes are made in their development (13). One of the most effective interventions or cares is multi-sensory stimulation for hospitalized premature infants with aim to simulate the intrauterine environment at first weeks of life in order to cause weight gain and development promotion in premature infants.

According to difference in total score of Ballard criteria and in the components of Ballard criteria including posture, are Recoil, popliteal angle, and heel to ear. The results of present study showed that neuromuscular development improved in the infants of intervention group. Multi-sensory stimulation has beneficial effect on neuromuscular development in premature infants. Multi-sensory stimulation can be an integral part of physical therapy in premature infants.

Short-term effects of multisensory stimulation in healthy preterm infants and, in preterm infants with periventricular leukomalacia was evaluated (14, 15). Significant effect of multi-sensory stimulation was not reported. In other words, duration, type of stimulation and neurodevelopmental scale may be different. Therefore, the results of this study cannot be compared with them. In another study, on the effect of multi-sensory stimulation on neurodevelopment, multisensory stimulation has a beneficial effect on neurodevelopment in preterm infants, which are consistent with the results of this study. 

There is no doubt that one of the tragedies of the world is the presence of people with physical or mental damage caused by preterm birth and many of these disorders are not diagnosed early, so it is difficult to predict late and long-term complications of infants discharged from ICU. Although neurodevelopment follow-up of these infants is an essential part of continuous evaluation and care of the infant, there is no standard process for this evaluation.

Developmental outcomes of very low birth weight infants were evaluated and a report on sensorineural and neurofunctional status of infants showed that such infants had more neurological problems and developmental delay compared with other infants (16). Moreover, another study on 7500 infants in Iran concluded that most common risk factors in infants' developmental disorder include prematurity, low birth weight, neonatal tetanus, hyaline membrane disease, mother's systemic infections during pregnancy, and severe neonatal hyperbilirubinemia (17).

There was a positive association between infant neurologic development and massage with human/social contact (18). “More recently, multisensory intervention in the form of play improved mental, motor, and social development in a group of children (age range of 6 months–2.5 yr) living in an orphanage in India” (19). 

The limitations of this study were as follows: Duration of follow up in the current study was very short. In addition, the effect of any of the stimulation in infants did not determine, future studies be done in this regard as separated and their results are compared with one another.

**In** **conclusion, **high-risk infants, children and preterm infants require special attention to have suitable development. These children are more suffering from development disorder or delay including motor, cognitive, speech, hearing, and vision disabilities compared with other children. Premature infants admitted in the neonatal intensive care unit undergone many invasive methods, and multi-sensory stimulation is an effective non-pharmacological method in the development of premature infants, and multisensory stimulation program improves neuromuscular development in the intervention group, it is recommended to perform this program as a standard care to reduce stress and improve neuromuscular development of premature infants.
